# *ccrAB*_Ent_ serine recombinase genes are widely distributed in the *Enterococcus faecium* and *Enterococcus casseliflavus* species groups and are expressed in *E. faecium*

**DOI:** 10.1099/mic.0.041491-0

**Published:** 2010-12

**Authors:** Eva Katrin Bjørkeng, Girum Tadesse Tessema, Eirik Wasmuth Lundblad, Patrick Butaye, Rob Willems, Johanna Ericsson Sollid, Arnfinn Sundsfjord, Kristin Hegstad

**Affiliations:** 1 Research Group for Host–Microbe Interactions, Department of Medical Biology, Faculty of Health Sciences, University of Tromsø, N-9037 Tromsø, Norway; 2 Reference Centre for Detection of Antimicrobial Resistance (K-res), Department of Microbiology and Infection Control, University Hospital of North-Norway, N-9038 Tromsø, Norway; 3 CODA-CERVA-VAR, Brussels, Belgium; 4 Department of Pathology, Bacteriology and Poultry Diseases, Faculty of Veterinary Medicine, Ghent University, Salisburylaan 133, 9820 Merelbeke, Belgium; 5 Department of Medical Microbiology, University Medical Centre Utrecht, Heidelberglaan 100, Rm G04.614, 3584 CX, Utrecht, The Netherlands

## Abstract

The presence, distribution and expression of cassette chromosome recombinase (*ccr*) genes, which are homologous to the staphylococcal *ccrAB* genes and are designated *ccrAB*_Ent_ genes, were examined in enterococcal isolates (*n*=421) representing 13 different species. A total of 118 (28 %) isolates were positive for *ccrAB*_Ent_ genes by PCR, and a number of these were confirmed by Southern hybridization with a *ccrA*_Ent_ probe (*n*=76) and partial DNA sequencing of *ccrA*_Ent_ and *ccrB*_Ent_ genes (*n*=38). *ccrAB*_Ent_ genes were present in *Enterococcus faecium* (58/216, 27 %), *Enterococcus durans* (31/38, 82 %), *Enterococcus hirae* (27/52, 50 %), *Enterococcus casseliflavus* (1/4, 25 %) and *Enterococcus gallinarum* (1/2, 50 %). In the eight other species tested, including *Enterococcus faecalis* (*n*=94), *ccrAB*_Ent_ genes were not found. Thirty-eight sequenced *ccrAB*_Ent_ genes from five different enterococcal species showed 94–100 % nucleotide sequence identity and linkage PCRs showed heterogeneity in the *ccrAB*_Ent_ flanking chromosomal genes. Expression analysis of *ccrAB*_Ent_ genes from the *E. faecium* DO strain showed constitutive expression as a bicistronic mRNA. The *ccrAB*_Ent_ mRNA levels were lower during log phase than stationary phase in relation to total mRNA. Multilocus sequence typing was performed on 39 isolates. *ccrAB*_Ent_ genes were detected in both hospital-related (10/29, 34 %) and non-hospital (4/10, 40 %) strains of *E. faecium*. Various sequence types were represented by both *ccrAB*_Ent_ positive and negative isolates, suggesting acquisition or loss of *ccrAB*_Ent_ in *E. faecium*. In summary, *ccrAB*_Ent_ genes, potentially involved in genome plasticity, are expressed in *E. faecium* and are widely distributed in the *E. faecium* and *E. casseliflavus* species groups.

## INTRODUCTION

The emergence of multidrug-resistant hospital-acquired *Enterococcus faecium* as one of the most important pathogens in the developed world has been a remarkable development in the last two decades (Leavis *et al.*, 2006; Werner *et al.*, 2003). Molecular epidemiological studies and comparative genomic hybridization analyses of *E. faecium* (Leavis *et al.*, 2007; Werner *et al.*, 2003) have revealed genotypic differences between hospital and community isolates (Leavis *et al.*, 2006). Mixed whole genome arrays demonstrated a distinct genetic make-up of hospital-associated *E. faecium* with more than 100 extra genes, possibly acquired by horizontal gene transfer (Leavis *et al.*, 2007). The *esp* virulence gene, located on a putative pathogenicity island, is one of the determinants acquired by hospital-associated *E. faecium*. These observations, as well as current multilocus sequence typing (MLST) data, strongly indicate that gene flux and recombination contribute significantly to diversification and adaptation of *E. faecium* (Leavis *et al.*, 2006; van Schaik *et al.*, 2010).

Recombinases facilitate the exchange of DNA fragments within and between bacteria and are thus pivotal in genome plasticity. Staphylococcal cassette chromosome (SCC) elements are vehicles for exchange of genetic information in staphylococci. These elements are characterized by the presence of terminal inverted repeats and unique recombinase genes, and are flanked by direct repeats (Ito *et al.*, 2001, 2004; Katayama *et al.*, 2003). So far, the major group of elements described are SCC*mec* I–VIII (International Working Group on the Classification of Staphylococcal Cassette Chromosome Elements, 2009) responsible for the spread of methicillin resistance between staphylococci. The movement of SCC elements is dependent on the gene products of the cassette chromosome recombinase genes (*ccr*), either the *ccrA*–*ccrB* complex or the single product of *ccrC* (Katayama *et al.*, 2000; Noto & Archer, 2006). These proteins are serine recombinases of the resolvase/invertase family which integrate the SCC element in a site-specific manner (Ito *et al.*, 1999). To our knowledge, *ccr* genes have only been reported in staphylococcal species.

Here, we report for the first time to our knowledge, the presence of *ccrAB* genes in enterococci, hereby designated *ccrAB*_Ent_, and show that they are expressed under standard *in vitro* growth conditions. Our analyses show that the *ccrAB*_Ent_ genes are widely distributed in *Enterococcus* species belonging to the *E. faecium* and *Enterococcus casseliflavus* species groups.

## METHODS

### Bacterial isolates.

A total of 421 *Enterococcus* isolates of 13 species from three continents (Europe, USA and Australia) were included in the study; *E. faecium* (*n*=216), *E. faecalis* (*n*=94), *E. durans* (*n*=38), *E. hirae* (*n*=52), *E. casseliflavus* (*n*=4), *E. avium* (*n*=4), *E. raffinosus* (*n*=3), *E. canintesti* (*n*=2), *E. canis* (*n*=2), *E. gallinarum* (*n*=2), *E. cecorum* (*n*=2), *E. asini* (*n*=1) and *E. dispar* (*n*=1). Among the 216 *E. faecium* isolates, 72 were of human origin of which 58 were clinical isolates. Among the 94 *E. faecalis* isolates, 13 were of human origin of which eight were clinical isolates. Other enterococcal species included were exclusively of animal origin (poultry, dog, bovine and pig). Six ATCC strains were also included. Isolates used for phylogenetic analyses, MLST and/or PCRs to link *ccrAB*_Ent_ with surrounding genes are displayed in Table 1.

The *E. faecium* ATCC 19434, *E. faecalis* ATCC 29212, *E. gallinarum* ATCC 49608, *E. faecalis* ATCC 19433 and *E. faecalis* ATCC 51575 were used as controls in species identification. All species were identified by *ddl* PCR (Dutka-Malen *et al.*, 1995) or tRNA intergenic spacer PCR (Baele *et al.*, 2000).

### Detection of *ccrAB*_Ent_ genes in the *E. faecium* DO genome by *in silico* analyses.

Preliminary sequence data of the *E. faecium* DO strain were obtained from The Joint Genome Institute (JGI) website at http://genome.jgi-psf.org/mic_home.html (version 08.06.04). Searches for homologous proteins were performed using blast 2.0 (http://www.ncbi.nlm.nih.gov/, on 8 February 2010) and fasta 33 (http://www.ebi.ac.uk/fasta33/, on 8 February 2010). Translation of coding sequences (CDSs) into amino acid sequences was done using ExPASy proteomic tools (http://au.expasy.org/tools/, on 8 February 2010).

For prediction of CDSs we used ORF finder (http://www.ncbi.nlm.nih.gov/gorf/gorf.html, on 8 February 2010), Gene Mark (v2.4) (Besemer & Borodovsky, 1999), FgenesB (http://www.softberry.com, on 2 August 2010) and artemis (Wellcome Trust Genome Campus, Hinxton, Cambridge, UK). Pairwise comparison and multiple sequence alignments were performed between the *E. faecium* CcrAB_Ent_ proteins and the previously identified four pairs of *Staphylococcus aureus* CcrABs (CcrAB1, CcrAB2, CcrAB3, CcrAB4; GenBank accession nos AB033763, D86934, AB037671 and AF411935) and CcrC (GenBank accession no. AB121219). Since *ccrB1* and *ccrB4* were truncated due to frame shift mutation, 1626 bp (*ccrB1*) and 1629 bp (*ccrB4*) ORFs were reconstituted by adding adenine to deleted positions in order to make the alignment better with the *Staphylococcus hominis ccr* sequence (GenBank accession no. AB063171) which has been fully sequenced (Ito *et al.*, 2001). The comparison of DNA sequences was performed in BioEdit v.7.0.5.3 (http://www.mbio.ncsu.edu/BioEdit/bioedit.html), while multiple alignments were done using clustal w (http://www.ebi.ac.uk/Tools/clustalw2/index.html) or T-Coffee (http://www.ebi.ac.uk/Tools/t-coffee/index.html).

The evolutionary relationships of CcrAB_Ent_, Ccr of staphylococci (deduced from *ccrA1*, *ccrA2*, *ccrA3*, *ccrA4*, *ccrB1*, *ccrB3*, *ccrB4* and *ccrC*), and three other site-specific recombinases (site-specific integrase of bacteriophage ϕ-FC1 found in *E. faecalis* and two site-specific recombinases from *Clostridium acetobutylicum* ATCC824) were further investigated. These were included because they have been part of previous similar analyses (Ito *et al.*, 2004) and because the *ccrA* and *ccrB*, as well as one of the recombinases from *C. acetobutylicum* (AE001437; locus tag no. CAC 2247), have been annotated as if they were DNA invertase Pin homologue proteins. The full-length *ccrB1* of NCTC10442 and *ccrB4* of HDE288 were reconstituted as described earlier (Ito *et al.*, 2004). A neighbour-joining tree was constructed using mega3 (Kumar *et al.*, 2004) by creating 2000 bootstrap replicates. Sites with gaps/missing data were excluded during analyses. Recombination within the sequenced regions of *ccrA*_Ent_ and *ccrB*_Ent_ was determined by phi test (Bruen *et al.*, 2006).

Protein structures were predicted using pstpred v2.4 (http://bioinf.cs.ucl.ac.uk/psipred/, on 17 December 2008) and the determinations of protein superfamilies were done using the HMM library, Genome assignment v1.65 (http://supfam.mrc-lmb.cam.ac.uk/SUPERFAMILY/, on 8 February 2010), InterProscan (http://www.ebi.ac.uk/InterProScan/, on 8 February 2010) and Pfam (http://pfam.sanger.ac.uk/, on 8 February 2010). The programs EditSeq and SeqMan (dnastar) were used for sequence analysis. To detect repeat sequences, Nucleic Acid Dot Plot (http://arbl.cvmbs.colostate.edu/molkit/dnadot/index.html, on 17 June 2010) and the Dotlet database (http://myhits.isb-sib.ch/cgi-bin/dotlet, on 8 February 2010) were used.

### DNA extraction, PCR amplification and DNA sequencing.

Bacterial DNA extraction for PCR analyses was performed manually by using the InstaGene matrix kit (Bio-Rad) or the GenoM-48 robotic workstation using GenoPrep DNA from blood, standard kit (Genovision). DNA for hybridization purposes was isolated using guanidium isothiocyanate (Dahl & Sundsfjord, 2003).

For long range PCR, 2 U DNA polymerase enzyme r*Tth* XL (Perkin Elmer) was used per reaction and 1.4 mM Mg(OAc)_2_ in a standard XL PCR mix, or a 0.7× *Pfu* Ultra mix (Stratagene) with 2.5 U *Pfu* Ultra polymerase per reaction. DNA sequencing was performed using BigDye 3.1 technology (Applied Biosystems). Real-time PCR was performed using ABI Prism 7300 real-time PCR system (PE Biosystems) and *Taq*Man universal mastermix (Applied Biosystems).

### Detection of *ccrAB*_Ent_ genes and PCR linkage to surrounding genes.

**Fig. 1.**
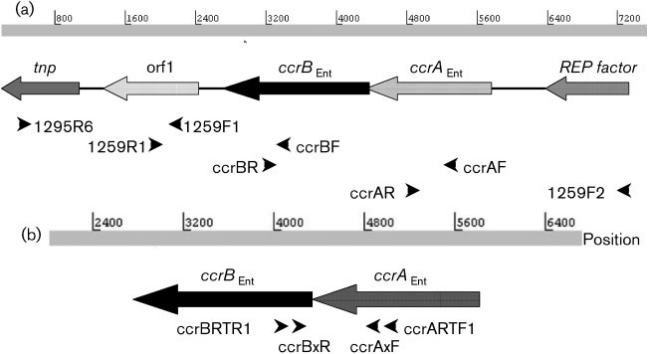
(a) Schematic presentation of the *ccrAB*_Ent_ region of *E. faecium* DO and the long-range PCRs used to link genes surrounding the *ccrA*_Ent_ and *ccrB*_Ent_ genes in *E. faecium*. (b) Schematic presentation of *ccrAB*_Ent_, indicating the positions of the PCR primers used for mRNA linkage. Linkage of *ccrA*_Ent_ and *ccrB*_Ent_ mRNAs was performed using combinations of primers ccrAxF/ccrBRTR1, ccrAxF/CcrBxR, CcrARTF1/ccrBRTR1 and CcrARTF1/ CcrBxR.

*ccrAB*_Ent_ genes were detected by PCR, using the primer pairs FA–RA and FB–RB, respectively (Table 2), and genes in selected isolates were detected by Southern hybridization and DNA sequencing. PCRs were also performed on 13 of 14 *ccrAB*_Ent_-positive *E. faecium* isolates selected for MLST as well as two *ccrAB*_Ent_-positive *E. faecium* animal isolates from Norway, to search for the presence and conservation of gene synteny in the surrounding genes (Table 2 and Fig. 1a). Primers and probes were designed using *E. faecium* DO sequences as template.

### Expression analysis of *ccrAB*_Ent_ genes by real-time quantitative PCR.

To analyse if *ccrAB*_Ent_ genes are expressed, *E. faecium* DO was grown aerobically in BHI broth at 37 °C for 18–24 h. Subsequently the culture was diluted 1 : 50 in BHI broth and grown with agitation to OD_600_ 0.7 or to stationary phase (grown overnight). The cell suspension was centrifuged and the cells were immediately frozen on dry ice or liquid nitrogen before adding an RNA stabilizing solution, RNA later (Ambion). Alternatively, RNA later or RNA protect (Qiagen) was added directly to the inoculum, according to the manufacturer's instructions. RNA extraction was performed by using the RNeasy mini kit (Qiagen) using a prolonged lysis step of 45 min with 10 mg lysozyme and 10 U mutanolysin in a total volume of 100 μl. On-column DNase treatment was performed according to the manufacturer's instructions. A successive removal of DNA was performed using Turbo DNase (Ambion) according to the manufacturer's instructions. RNA integrity was determined by agarose gel electrophoresis. Reverse transcription of the total RNA was performed using the ABRTR1 primer and the High Capacity cDNA Reverse Transcription kit (Applied Biosystems) or Superscript III RNase H-reverse transcriptase (Invitrogen). Real-time PCR was performed on the cDNA using primers ccrAFre, ccrARre, ccrBFre, ccrBRre, recAFre, recARre, pbp5Fre, pbp5Rre, adkFre and adkRre, and probes ccrA_Ent_, ccrB_Ent_, recA, pbp5 and adk (Table 2). Expression of *ccrAB*_Ent_ genes was compared with the expression of *recA*, *pbp5* and *adk*. Ten-fold serial dilutions of *E. faecium* DO genomic DNA were used to make standard curves to determine PCR efficiency, using the equation: E=10^(−1/slope)^−1. The PCR efficiencies ranged from 88 to 104 % in one assay and 99 to 100 % in a second assay and were considered similar enough to be able to compare only *C_t_* (threshold cycle) values for a semiquantitative relative measurement of expression. The expression experiments were performed in three triplicates; a no template control (NTC) and a minus reverse transcriptase control (−RT) was included in each assay. The −RT controls were in the range of an acceptable difference from the cDNA expression analysis (>5*C_t_* difference).

### Analysis of *ccrAB*_Ent_ mRNA linkage by RT-PCR.

RNA isolation was performed as described above. RNA was treated with the DNA-free kit (Ambion). Reverse transcription of total RNA was performed with SuperScript III reverse transcriptase (Invitrogen) using primers CcrBRTR1 or CcrBxR. RT-PCR without reverse transcriptase was performed on total RNA to check for DNA contamination. Linkage of *ccrA*_Ent_ and *ccrB*_Ent_ mRNAs as a bicistronic mRNA was analysed by PCRs on cDNAs using primers located in *ccrA*_Ent_ (CcrARTR1 and CcrAxF) and *ccrB*_Ent_ (CcrBRTR1 and CcrBxR) (Fig. 1b and Table 2).

### Southern blot hybridization analyses.

RFLP with *Xba*I (Promega) was performed on total genomic DNA for selected *E. faecium* isolates (DO, TUH7-55, E0470, E0745, E1304 and E1293). PFGE of *Sma*I-digested DNA from 76 *E. faecium* isolates was performed as described by Dahl *et al.* (1999). DNA fragments separated by gel electrophoresis were transferred to a positively charged nylon membrane (Boehringer) by vacuum blotting using a Vacugene XL system (Amersham Biosciences). Southern blot hybridization was performed with a DIG-labelled (Boehringer) *ccrA*_Ent_ probe based on *E. faecium* DO.

### MLST.

MLST was performed on a subset of isolates using the following primers: adk1n, adk2n, atpA1n, atpA2n, ddl1, ddl2, gdh1, gdh2, gyd-1, gyd2, pstS1n, pstS2, purK1n and purK2n (Homan *et al.*, 2002).

## RESULTS AND DISCUSSION

### *ccrAB*_Ent_ sequences in the *E. faecium* DO genome

Genes similar to the *ccrA* and *ccrB* genes of *S. aureus* (GenBank accession no. D86934) were identified in the draft sequence of the *E. faecium* DO genome. blast searches indicated two CDSs (locus tag nos 2319 and 2398) in *E. faecium* DO contig 655 (version 08.06.04) similar and in an identical order to the staphylococcal *ccrA* and *ccrB*. They were named *ccrA*_Ent_ and *ccrB*_Ent_. No available reports have previously shown *ccrA*_Ent_ and *ccrB*_Ent_ genes in enterococci. The *ccrA*_Ent_ and *ccrB*_Ent_ CDSs are 1374 bp and 1638 bp in size, respectively. The two *ccrAB*_Ent_ genes in *E. faecium* DO were similar in length to the staphylococcal *ccrAB2* (Katayama *et al.*, 2000).

The *ccrAB*_Ent_ gene synteny was confirmed to be the same as in staphylococci (Katayama *et al.*, 2000) for 14 of 15 *E. faecium* isolates by linkage PCR (Table 3). No available results have previously shown whether staphylococcal *ccrA* and *ccrB* genes are transcribed as separate units or as a bicistronic mRNA. RT-PCR analysis of total RNA from *E. faecium* DO revealed that the *ccrAB*_Ent_ genes were transcribed as a bicistronic mRNA, confirming the bioinformatics results. Knowing the function of *ccrAB* in staphylococci, we hypothesize that *ccrAB*_Ent_ genes in enterococci might be part of a larger integrative genetic element in *E. faecium*. The GC content of *E. faecium* DO contig 655 (35 %), the *ccrAB*_Ent_ CDSs (35 %) and the whole genome (38 %) is not substantially different. No putative termini (repeats) were identified in contig 655 by nucleic acid dot plot or DotLet analyses. Thus it was not possible to identify a putative integrative element. The genome sequence of contig 655 is limited to the *tnp* transposase determinant (Fig. 1a) at the left side and it has not been possible to identify the continuation of this sequence in another DO contig. The sequence at the other side of *ccrAB*_Ent_ also contains a lot of putative transposases (belonging to several insertion sequence families) in addition to hypothetical proteins (http://maple.lsd.ornl.gov/cgi-bin/JGI_microbial/contig_viewer.cgi?org=efae&chr=08jun04&contig=Contig655&sort=left_bp, on 21 June 2010) which may well be part of an integrative element.

Pairwise comparison and multiple sequence alignments were performed between the *E. faecium* CcrAB_Ent_ proteins and the Ccr proteins of *S. aureus*. The similarities of CcrA and CcrB between *E. faecium* and *S. aureus* N315 were 55 and 69 %, respectively. The N-terminal resolvase and recombinase domains, as well as the predicted catalytic serine residue of the recombinase active site were highly conserved between the *Staphylococcus* and *Enterococcus* CcrAB proteins. Moreover, the *Enterococcus* CcrB_Ent_ was predicted to include an Ogr/delta-like domain (a phage transcription activator). Two algorithms, Pfam and ProScan, predicted both the resolvase and recombinase domains in the examined Ccr protein sequences (Supplementary Table S1, available with the online version of this paper).

**Fig. 2.**
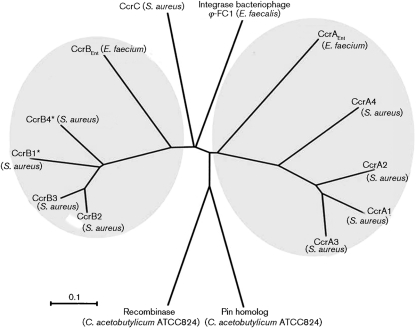
Phylogram for CcrA_Ent_, CcrB_Ent_, other Ccrs, and three site-specific recombinase proteins. The deduced amino acid sequences of the following genes were used: *ccrA1* and ccrB1* (from NCTC10442, GenBank accession no. AB033763); *ccrA2* and *ccrB2* (from N315, GenBank accession no. D86934); *ccrA3* and *ccrB3* (from 85/2082, GenBank accession no. AB037671); *ccrA4* and ccrB4* (from HDE288, GenBank accession no. AF411935); *ccrC* [from JSCC 3624 (WIS), GenBank accession no. AB121219]; site-specific integrase (from phi-FC1, GenBank accession no. AF124258); and two site-specific recombinases (from *C. acetobutylicum* ATCC824, GenBank accession no, AE001437, locus tag nos CAC 1228 and CAC 2247). The scale bar indicates genetic distance in substitutions per site. The Ccr clusters are circled. The amino acid sequences were aligned using T-Coffee. The neighbour-joining phylogenetic tree was constructed with mega3 from 2000 bootstrap replicates using the P-distance model. The dataset consisted of 447 amino acids with 37 parsimony-informative sites for CcrA and 547 amino acids with 50 parsimony-informative sites for CcrB.

The evolutionary relationships of CcrAB_Ent_, Ccr of staphylococci and three other site-specific recombinases were further investigated. The phylogenetic analyses revealed an evolutionary relationship between CcrA_Ent_ and CcrB_Ent_ from enterococci and the staphylococcal CcrAB cluster (Fig. 2). However, the low identity score between the enterococcal and staphylococcal proteins does not support a recent horizontal transfer of the *ccr* genes between these species.

### *ccrAB*_Ent_ genes are expressed in *E. faecium*

Analyses of *ccrAB*_Ent_ gene expression were performed during both the exponential and stationary phase of *E. faecium* DO grown in rich medium. Both genes were expressed in approximately the same amounts in exponential phase. *ccrAB*_Ent_ genes were expressed >70-fold lower than the *pbp5*, *recA* and *adk* genes (Supplementary Fig. S1). The mRNA abundance of *ccrAB*_Ent_ was lower in stationary phase than in exponential phase.

### *ccrAB*_Ent_ genes are dispersed among *Enterococcus* species belonging to the *E. faecium* and *E. casseliflavus* species groups

Of a total of 421 enterococcal isolates, 118 (28 %) were positive for *ccrAB*_Ent_ genes in five species by PCR; *E. faecium* (58/216, 27 %), *E. durans* (31/38, 82 %), *E. hirae* (27/52, 50 %), *E. casseliflavus* (1/4, 25 %) and *E. gallinarum* (1/2, 50 %) (Table 1). One *E. hirae* isolate was positive by PCR for *ccrB*_Ent_ only. Eight other species including *E. faecalis* were negative for *ccrAB*_Ent_ (data not shown).

A blast search for the *ccrAB*_Ent_ genes and the surrounding regions against *Enterococcus* strains revealed the presence of *ccrAB*_Ent_ in *E. faecium* E1071, 1,231,408 and C68 (http://www.ncbi.nlm.nih.gov/genomes/geblast.cgi?gi=6512#SearchSet, on 21 June 2010) and no *ccrAB*_Ent_ sequence or protein matches with high identity scores in other available *Enterococcus* genomes (http://www.ncbi.nlm.nih.gov/sutils/genom_table.cgi, on 21 June 2010). *E. faecium* E1071 and *E. faecium* 1,231,408 showed sequence similarity with the DO sequence in parts of the hypothetical protein, *ccrB*_Ent_ and parts of *ccrA*_Ent_. *E. faecium* C68 showed similarity with DO in parts of the hypothetical protein, both *ccrA*_Ent_ and *ccrB*_Ent_ and parts of the replication initiation factor (REP factor).

**Fig. 3.**
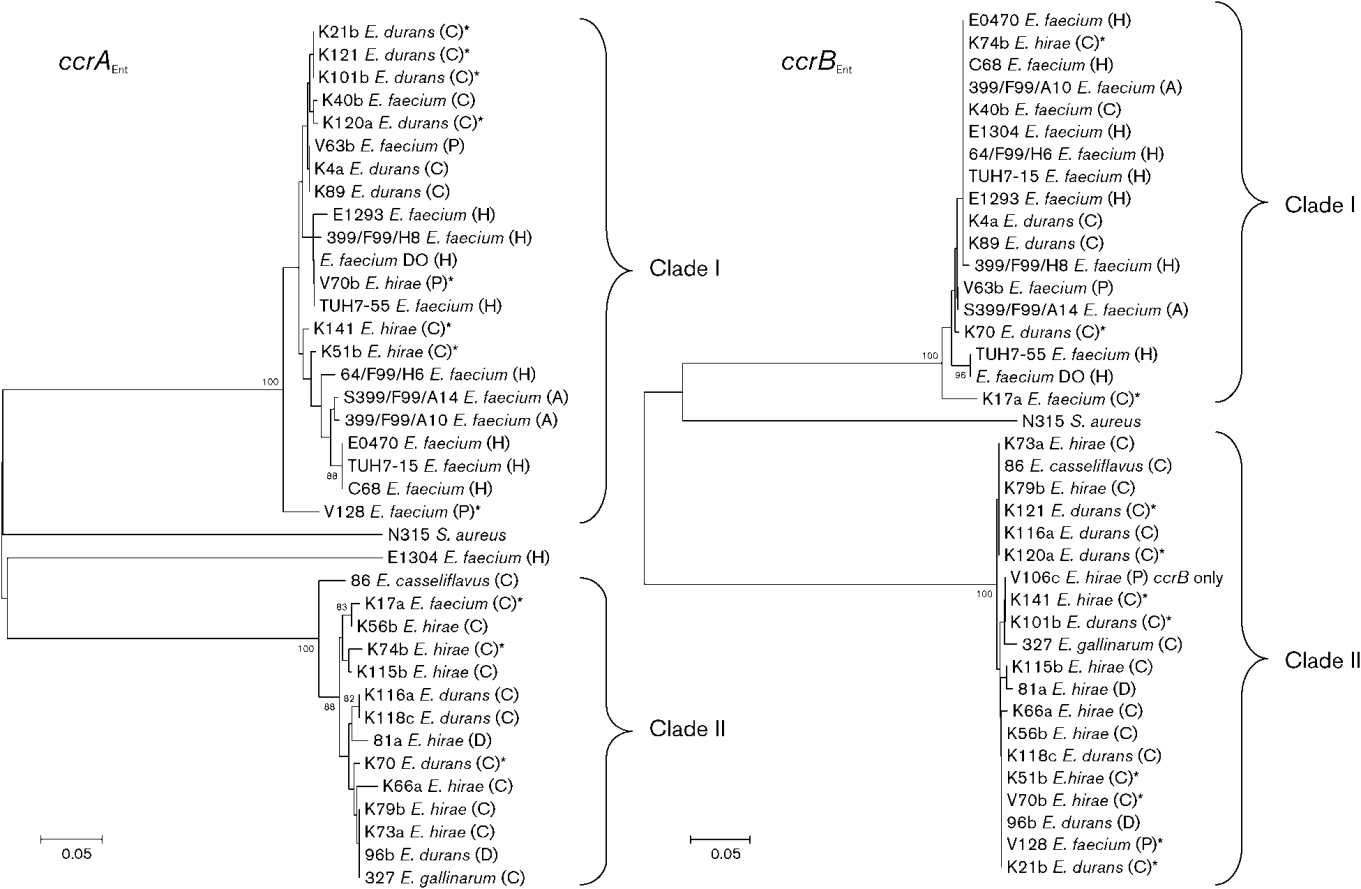
Phylogram for *ccrA*_Ent_ and *ccrB*_Ent_ genes. The nucleotide sequences for *ccrA*_Ent_ and *ccrB*_Ent_ genes from *E. faecium* (*n*=15), *E. hirae* (*n*=10/11), *E. durans* (*n*=10), *E. casseliflavus* (*n*=1) and *E. gallinarum* (*n*=1) were used (GenBank accession nos FJ572967–FJ573039). Upper case letters in parentheses represent the origin of the isolate: (C), chicken; (H), human; (D), dog; (P), pig; and (A), unknown animal origin. Outgroups are represented by *S. aureus* N315 *ccrA* and *ccrB* (GenBank accession no. D86934). The asterisks indicate isolates in which *ccrA*_Ent_ and *ccrB*_Ent_ belong to different clades. All sequences were aligned using clustal w. The neighbour-joining phylogenetic tree was made with mega4.0 using 2000 bootstrap replicates and the P-distance model. Bootstrap values higher than 80 % are shown at the branches. The scale bar indicates genetic distance in substitutions per site. The two main clades of *ccrA*_Ent_ and *ccrB*_Ent_ are indicated. The dataset consisted of 547 nt with 494 parsimony-informative sites for *ccrA*_Ent_ and 513 nt with 227 parsimony-informative sites for *ccrB*_Ent_.

*ccrAB*_Ent_ gene sequences (GenBank accession nos FJ572967–FJ573039) from *E. faecium* (*n*=14), *E. hirae* (*n*=10 for *ccrA*_Ent_ and 11 for *ccrB*_Ent_), *E. durans* (*n*=10), *E. gallinarum* (*n*=1) and *E. casseliflavus* (*n*=1) isolates were aligned and a neighbour-joining phylogenetic tree was made with 2000 bootstrap replicates using the P-distance model (Fig. 3). The *ccrAB*_Ent_ genes both clustered into two major clades represented by the majority of *E. faecium* (clade I) and *E. hirae* (clade II) isolates, respectively. With 7 of 10 isolates clustering in clade II, *E. hirae* appears to be slightly more dispersed between the two *ccrA*_Ent_ clades. *ccrAB*_Ent_ from the *E. gallinarum* and *E. casseliflavus* isolates clustered in clade II with the majority of *ccrAB*_Ent_ from the *E. hirae* isolates. In *E. durans*, 6 of 10 *ccrA*_Ent_ genes clustered in clade I, while 7 of 10 *ccrB*_Ent_ clusters were in clade II. Except for *ccrA*_Ent_ from *E. faecium* E1304, the *ccrAB*_Ent_ genes of the human isolates clustered in clade I whereas the animal isolates were found in both clades. Incongruence between *ccrA*_Ent_ and *ccrB*_Ent_ phylogenies within an isolate was noted for 11 isolates, all of animal origin (Fig. 3, isolates marked with asterisks). Phi tests revealed no statistically significant evidence for recombination within the sequenced regions of the *ccrA*_Ent_ and *ccrB*_Ent_ genes. However, the incongruence suggests recombination of the *ccr*_Ent_ genes outside the sequenced regions of the two genes. Incongruence between these genes has also been seen for *S. aureus* (Ito *et al.*, 2004).

*ccrAB*_Ent_ genes were only found in isolates belonging to the *E. faecium* and *E. casseliflavus* species groups that belong to the same tree branch in phylogenetic trees based on enterococcal 16S and *sodA* gene diversity (Devriese *et al.*, 1993; Poyart *et al.*, 2000). The absence of *ccrAB*_Ent_ in the other species could be explained by the low number of isolates tested, except for *E. faecalis*, or by a lack of integration sites recognized by *ccrAB*_Ent_ in the strains not belonging to the *E. faecium* or *E. casseliflavus* groups. Alternatively, their *ccrAB*_Ent_ genes may exhibit such a low sequence identity to the *ccrAB*_Ent_ genes identified in this study that they are missed using the PCR and hybridization conditions used in the present study.

### Variations of the *ccrAB*_Ent_ genes and the surrounding region between selected *E. faecium* isolates

PFGE analysis and Southern hybridization of 76 *E. faecium* isolates with the *ccrA*_Ent_ probe confirmed the PCR results. One *ccrA*_Ent_ PCR-negative strain (399/F98/A1) was *ccrA*_Ent_-positive by Southern blot hybridization (data not shown) indicating that sequence diversity affects PCR amplification. Also, *Xba*I analyses of *ccrA*_Ent_ and *ccrB*_Ent_ genomic regions revealed heterogeneity and only one copy of *ccrA*_Ent_. The *ccrA*_Ent_ probe hybridized to an approximately 10 kb fragment in DO, TUH7-55, E1304 and E1293 isolates; however, the *ccrA*_Ent_-positive fragment of E0470 and E0745 was approximately 24 kb (data not shown). To investigate this in more detail, the presence of *ccrAB*_Ent_ flanking genomic genes identified in the DO genome was determined by multiple PCRs in 15 *ccrAB*_Ent_-positive and 16 *ccrAB*_Ent_-negative isolates (Fig. 1a). Examinations of the *ccrAB*_Ent_ surrounding region in several isolates showed a variable pattern of the *ccrAB*_Ent_ flanking sequences with hospital-associated isolates showing most sequence similarity with the DO sequence (Table 3). All 31 isolates were positive for the *tnp* gene-specific PCR (*tnp* belongs to the IS*30* family) as well as for orf1 PCR and three *ccrAB*_Ent_-positive isolates of different sequence types (STs) were also positive for the REP factor gene PCR. This REP factor gene harbours a REP_*trans* domain belonging to superfamily pfam02486. This family represents probable topoisomerases that makes a sequence-specific single stranded nick in the origin of replication. Plasmid REPs, phage REPs (RstAs) and transposon REPs (Cro/CI transcriptional regulators) belong to this family. Long-range PCRs confirmed linkage of these genes with *ccrAB*_Ent_ and conservation of gene synteny surrounding *ccrAB*_Ent_ with the exception of isolates 64/F99/H6, 399/F99/A10, 399/F99/H8, and S399/F99/A14, for which linkage of *tnp*–orf1 and orf1–*ccrB*_Ent_ was not confirmed. Furthermore, a *ccrB*_Ent_–*ccrA*_Ent_ linkage was not shown in 64/F99/H6 (Table 3 and Fig. 1a). The inability to link genes that were positive on gene-specific PCRs may indicate that the region between these genes is larger than expected or that the specific genes are located at other regions in the genome. The transposase of the IS*30* family is, for instance, located at more than one site in *E. faecium* DO. Annotation of contig 655 (http://maple.lsd.ornl.gov/cgi-bin/JGI_microbial/contig_viewer.cgi?org=efae&chr=08jun04&contig=Contig655&sort=left_bp, on 21 June 2010) also indicates that the *ccrAB*_Ent_ genes are located in a region containing several transposases. The regions surrounding *ccrAB* in staphylococci contain highly variable genes encoding ORFs of unknown functions. These variable regions are called J1 and J2, and variations in these regions are used to define the SCC*mec* subtypes (International Working Group on the Classification of Staphylococcal Cassette Chromosome Elements, 2009), and so our results from enterococci are in line with these observations of highly variable regions surrounding *ccrAB* in staphylococci. CcrA and CcrB have roles in the excision and integration of SCC*mec* in staphylococci (Wang & Archer, 2010) and we have showed that the *ccrAB*_Ent_ genes are expressed in *E. faecium* DO. It has been postulated that SCC may carry the genes conferring methicillin resistance but may also enable genetic exchange of other genes among staphylococcal species (Katayama *et al.*, 2003). However, to our knowledge, no studies have provided direct experimental evidence for intercellular transfer of SCC between staphylococci.

DNA sequencing of the *ccrAB*_Ent_, *tnp* and *orf1* of the 15 *ccrAB*_Ent_-positive isolates showed 94–100 % and 96–100 % sequence identity in *ccrA*_Ent_ and *ccrB*_Ent_ genes (GenBank accession nos FJ572978–FJ572981, FJ572997–FJ573001, FJ573014–FJ573018, FJ573032–FJ573036), respectively, while sequences of *orf1* and *tnp* were 100 % identical in all isolates (data not shown). According to Hanssen *et al.* (2004), up to 4 % variation within the *ccrAB* genes has been observed for a given staphylococcal species. The *ccrAB* genes found in SCC*mec* types II and IV can vary up to 5 % at the nucleotide level (Noto & Archer, 2006). Since both *ccrAB*_Ent_ genes and the staphylococcal *ccrAB* genes show sequence variations within the recombinase genes, which have the same gene synteny and variable surrounding regions, we hypothesize that they may have similar functions in contributing to excision and integration of surrounding genes within the genome and possibly also mobilization of surrounding genes between cells.

### Investigation of possible association between *ccrAB*_Ent_ and ST within *E. faecium* of human origin

MLST analyses of *E. faecium* isolates (*n*=39) revealed that the *ccrAB*_Ent_ genes are dispersed among different STs (Table 1). Ten of 29 (34 %) hospital-related *E. faecium* isolates were *ccrAB*_Ent_-positive, while 4 of 10 (40 %) non-hospital-related isolates were positive. Furthermore, specific STs within hospital-related strains were represented by both *ccrAB*_Ent_-positive and -negative isolates (Table 1), suggesting that *ccrAB*_Ent_ genes are acquired and not a part of the core genome.

### Concluding remarks

Cassette chromosome recombinases may be important in recombination and genome plasticity in enterococci. Expression analyses indicate that the recombinase genes are active in *E. faecium* DO and thus, may play a role in the recombination or movement of genetic elements. Further investigation of the *ccrA*_Ent_ and *ccrB*_Ent_ will be essential to reveal the contribution of these genes for recombination and mobilization events in enterococci.

## Figures and Tables

**Table 1.** *Enterococcus* isolates selected for MLST typing, phylogenetic analyses and/or PCRs to link *ccrAB*_Ent_ with surrounding genes Type of vancomycin resistance, ST or hospital-related ST (CC17 genogroup), and the presence of *ccrAB*_Ent_ genes is shown. nd, not determined; −, negative.

**Isolate name**	**Origin country/region**	**Sample source**	Epidemiology*	***van* type**	**ST**	***ccrAB*_Ent_**	**Reference/source**
***E. faecium***							
C68	USA/Ohio	Human faeces	CI	*vanB*	16 (CC17)	AB	Carias *et al.* (1998)
E0470	Netherlands/Amsterdam	Human blood	HO	*vanA*	16 (CC17)	AB	Willems *et al.* (2005)
E0734	Netherlands/Amersfoort	Hospital faeces	HO	*vanA*	16 (CC17)	AB	Willems *et al.* (2005)
E0745	Netherlands/Utrecht	Human faeces	HO	*vanA*	16 (CC17)	AB	Willems *et al.* (2005)
TUH7-15	USA	Human blood	HO	*vanB*	16 (CC17)	AB	Dahl *et al.* (1999)
E0510	Australia/Melbourne	Human blood	HO	*vanB*	17 (CC17)	−	Willems *et al.* (2005)
TUH2-18	Norway/Bergen	Human urine	HO	*vanB*	17 (CC17)	−	Dahl *et al.* (1999)
TUH2-19	Norway/Bergen	Human wound	HO	*vanB*	17 (CC17)	−	Dahl *et al.* (1999)
TUH7-55	Germany	Human urine	CI	*vanB*	17 (CC17)	AB	Dahl *et al.* (1999)
DO (TX0016)	USA/Houston	Human blood	CI	−	18 (CC17)	AB	Arduino *et al.* (1994)
E1652	Netherlands/Amersfoort	Human faeces	HO	*vanA*	18 (CC17)	−	Willems *et al.* (2005)
E1406	Spain/Madrid	Human blood	HP	nd	63 (CC17)	−	T M. Coque/R. Willems
E1392	Great Britain/Centre H	Human	HP	nd	64 (CC17)	−	N. Woodford/R. Willems
E1181	Austria/Linz	Human blood	HP	nd	78 (CC17)	−	ENARE/R. Willems
E1186	Germany	Human blood	HP	nd	78 (CC17)	−	ENARE/R. Willems
E1321	Italy/Rome	Human catheter	HP	nd	78 (CC17)	−	L. Baldassarri/R. Willems
E1644	Germany/Freiburg	Human catheter urine	HP	nd	78 (CC17)	−	D. Jonas/R. Willems
E0333	Israel/Centre1	Human blood	HP	nd	80 (CC17)	−	R. Schouten/R. Willems
E1775	Belgium	Pig faeces		nd	121 (CC17)	AB	E. de Leener/R. Willems
E1173	Portugal/Coimbra	Human wound	CI	*vanA*	125 (CC17)	−	Willems *et al.* (2005)
E1304	Portugal/Coimbra	Human blood	CI	*vanA*	132 (CC17)	AB	Willems *et al.* (2005)
E1762	Australia/Perth	Human	Hospital survey	nd	174 (CC17)	−	W. Grubb/R. Willems
U0105	Netherlands	Human blood	HP	nd	267 (CC17)	−	A. Troelstra/R. Willems
3332	USA/Ohio	Human	HO	*vanB*	308 (CC17)	AB	Carias *et al.* (1998)
TUH4-65	USA	Human	CI	*vanB*	313 (CC17)	−	Dahl *et al.* (1999)
E0125	Netherlands/Rotterdam	Human bile	CI	*vanA*	5	−	Willems *et al.* (2005)
399/F98/H2	Norway/Østfold	Human faeces	CS	*vanA*	8	−	Johnsen *et al.* (2005)
64/3	Germany	Human faeces	HP	−	21	−	Werner *et al.* (2003)
E0073	Netherlands/Rotterdam	Human faeces	CI	*vanA*	22	−	Willems *et al.* (2005)
S399/S99/H5	Norway/Østfold	Human faeces	CS	−	48	−	Johnsen *et al.* (2005)
64/F98/H1	Norway/Østfold	Human faeces	CS	*vanA*	48	−	Johnsen *et al.* (2005)
E1293	Italy/Geneva	Human blood	CI	−	50	AB	Willems *et al.* (2005)
E1626	Netherlands	Human peritoneal fluid	CI	−	92	−	Willems *et al.* (2005)
BM4105-RF	France	Human faeces	Wild strain	−	172	−	Poyart & Trieu-Cuot (1994)
399/F99/H8	Norway/Østfold	Human faeces	CS	*vanA*	195	AB	Johnsen *et al.* (2005)
64/F99/H6	Norway/Østfold	Human faeces	CS	*vanA*	246	AB	Johnsen *et al.* (2005)
399/F99/A10	Norway/Østfold	Animal faeces	CS	*vanA*	310	AB	Johnsen *et al.* (2005)
399/F98/A1	Norway/Østfold	Animal faeces	CS	*vanA*	311	−†	Johnsen *et al.* (2005)
S399/F98/H3	Norway/Østfold	Human faeces	CS	−	312	−	Johnsen *et al.* (2005)
K17a	Belgium	Chicken		nd	nd	AB	P. Butaye
K40b	Belgium	Chicken		nd	nd	AB	P. Butaye
S399/S99/A4	Norway/Østfold	Animal faeces	CS	−	nd	AB	Johnsen *et al.* (2005)
S399/F99/A14	Norway/Østfold	Animal faeces	CS	−	nd	AB	Johnsen *et al.* (2005)
V63b	Belgium	Pig		nd	nd	AB	P. Butaye
V128	Belgium	Pig		nd	nd	AB	P. Butaye
***E. durans***							
K101b	Belgium	Chicken		nd	nd	AB	P. Butaye
K4a	Belgium	Chicken		nd	nd	AB	P. Butaye
K21b	Belgium	Chicken		nd	nd	AB	P. Butaye
K70	Belgium	Chicken		nd	nd	AB	P. Butaye
K89	Belgium	Chicken		nd	nd	AB	P. Butaye
K116a	Belgium	Chicken		nd	nd	AB	P. Butaye
K118c	Belgium	Chicken		nd	nd	AB	P. Butaye
K120a	Belgium	Chicken		nd	nd	AB	P. Butaye
K121	Belgium	Chicken		nd	nd	AB	P. Butaye
96b	Belgium	Dog		nd	nd	AB	P. Butaye
***E. hirae***							
K51b	Belgium	Chicken		nd	nd	AB	P. Butaye
K56b	Belgium	Chicken		nd	nd	AB	P. Butaye
K66a	Belgium	Chicken		nd	nd	AB	P. Butaye
K73a	Belgium	Chicken		nd	nd	AB	P. Butaye
K74b	Belgium	Chicken		nd	nd	AB	P. Butaye
K79b	Belgium	Chicken		nd	nd	AB	P. Butaye
K115b	Belgium	Chicken		nd	nd	AB	P. Butaye
K141	Belgium	Chicken		nd	nd	AB	P. Butaye
V70b	Belgium	Pig		nd	nd	AB	P. Butaye
V106c	Belgium	Pig		nd	nd	B	P. Butaye
81a	Belgium	Dog		nd	nd	AB	P. Butaye
***E. casseliflavus***							
86	Belgium	Chicken		nd	nd	AB	P. Butaye
***E. gallinarum***							
327	Belgium	Chicken		nd	nd	AB	P. Butaye

*CI, Clinical isolate; HO, hospital outbreak; HP, hospitalized patient; CS, community survey. Details are provided for human isolates only. †Positive for *ccrA*_Ent_ only by Southern hybridization.

**Table 2.** Oligonucleotides used for expression analyses of *ccrAB*_Ent_ genes and for detection/characterization of the *ccrAB*_Ent_ region and detection of enterococcal virulence genes

**Purpose**	**Target gene**	**Primer name**	**Sequence (5′–3′)**	**Product size (bp)**	**Reference**
Expression study					
	*ccrA*_Ent_	ccrAFre	AACGATTGACGCAACAAAAGCT	129	This study
		ccrARre	CGCCATAGTACAATGGATTTTTTAGGATAT		
		ccrA_Ent_ probe	TCCGCGAACGTCCTTT		
	*ccrB*_Ent_	ccrBFre	TTTTCTACCACGGCAGTCAAAGAT	68	This study
		ccrBRre	CAATTGATGTAGCGCGCATATTCTA		
		ccrB_Ent_ probe	ACCCTGCATAAATTTT		
	*recA*	recAFre	GATTCAGTTGCTGCTTTAGTTCCA	72	This study
		recARre	CTTGTAACCCGACATGTGAGTCA		
		recA probe	TTCGCCGTCGATTTC		
	*pbp5*	pbp5Fre	GATCTGGTTTGGAAATGGCTTTTGA	79	This study
		pbp5Rre	CACCGTCTGTATCTGTGATGCTTAA		
		pbp5 probe	TCCCACGAAGATCCTT		
	*adk*	adkFre	CCACGTACGCTAGATCAAGCAA	85	This study
		adkRre	CATGGATATCGATGACAGCATCAATTTT		
		adk probe	ATTGCGTCCAGAGCTT		
*ccrAB*_Ent_ linkage of RT-PCR product and RT-PCR control					
	*ccrAB*_Ent_	ccrAxF	CGAAAAGCGAAAAGATGAAAAACACAAAGT		This study
		ccrARTR1	ACCTCGATCCGACAAACATGGTCACATAAC	222	
		ccrBxR	ACATAGCCTAAACGTCGTCCACCTG	625	
		ccrBRTR1	TAACCCCACATCATATCGCAACAGTTCCTC	801	
PCRs to sequence a part of the genes/CDSs					
	*ccrA*_Ent_	ccrAF	GAAATATGAACAAATTCCCCAACG	451	This study
		J03/252ccrARB	TTGAAAAATATAGCGAACAATCC		
	*ccrB*_Ent_	J03/252ccrBF	TCGGAATAAAGGAGCAAGTGTG	525	This study
		ccrBR	GCAGGCGTGAATTTCATTGTA		
Detection in the early phase of the study (later changed for new primers)					
	*ccrA*_Ent_	ccrAF	GAAATATGAACAAATTCCCCAACG	1242	This study
		ccrAR	CGGAAGTAAATCCCACAGACT		
	*ccrB*_Ent_	ccrBF	GGAACCATCGTTTTGATCTACTAG	1321	This study
		ccrBR	GCAGGCGTGAATTTCATTGTA		
New primers used for detection					
	*ccrA*_Ent_	FA	CCATATGGGTATCGTTTAGTGA	453	This study
		RA	AGCTTCGGTCGGTACAATGAT		
	*ccrB*_Ent_	FB	ATTTGTCGCCGACCGATTAAAG	390	This study
		RB	ACGATACAAGGCTTTGAYTTGCT		
Others					
	orf1	1259F1	ATTTGTTACTGAATCCAGTGCTTACTC	873	This study
		1259R1	CAATGTTATTCTGCTTGAACTTGACC		
	REP factor	1259F2	GCTAGGAGTACAAAATATCCAACGC	721	This study
		1259R2	CTGAATAATTCTCCGTATGAGAGCG		
	*tnp*	1259F6	CGAAGCAGCTTAAACGTGGAC	759	This study
		1259R6	GGATATGGTTTCTTTTGGACGC		

**Table 3.** Long-range linkage PCR results for the *ccrA*_Ent_ and *ccrB*_Ent_ chromosomal region among 15 *ccrAB*_Ent_-positive *E. faecium* isolates +, Positive; −, negative; na, not applicable (one of the genes/CDSs not present); nd, not determined.

**Isolate**	ST*	**Long-range linkage PCRs**			
		***tnp*–orf1**	**orf1–*ccrB*_Ent_**	***ccrB*_Ent_–*ccrA*_Ent_**	***ccrA*_Ent_–*REP factor***
DO	**18**	+	+	+	+
E1304	**132**	+	+	+	+
TUH7-55	**17**	+	+	+	+
3332	**308**	+	+	+	na
C68	**16**	+	+	+	na
E0470	**16**	+	+	+	na
E0734	**16**	+	+	+	na
E0745	**16**	+	+	+	na
TUH7-15	**16**	+	+	+	na
64/F99/H6	48	−	−	−	na
399/F99/A10	310	−	−	+	na
399/F99/H8	195	−	−	+	na
E1293	50	na	na	+	na
S399/F99/A14	nd	−	−	+	na
S399/S99/A4	nd	na	na	+	na

*STs in bold belong to the CC17 genogroup.

## Abbreviations

- **s:** ATCC, American Type Culture Collection
- CDSs, coding sequences
- JGI, Joint Genome Institute
- MLST, multilocus sequence type
- ST, sequence type
- SCC, staphylococcal cassette chromosome
